# Multimodal Percutaneous Thermal Ablation of Small Hepatocellular Carcinoma: Predictive Factors of Recurrence and Survival in Western Patients

**DOI:** 10.3390/cancers12020313

**Published:** 2020-01-29

**Authors:** Margaux Hermida, Christophe Cassinotto, Lauranne Piron, Serge Aho-Glélé, Chloé Guillot, Valentina Schembri, Carole Allimant, Samir Jaber, Georges-Philippe Pageaux, Eric Assenat, Boris Guiu

**Affiliations:** 1 Department of Radiology, St-Eloi University Hospital, 34980 Montpellier, France; m-hermida@chu-montpellier.fr (M.H.); c-cassinotto@chu-montpellier.fr (C.C.); l-piron@chu-montpellier.fr (L.P.); chloe-guillot@chu-montpellier.fr (C.G.); v-schembri@chu-montpellier.fr (V.S.); carole.allimant@gmail.com (C.A.); 2 Department of Epidemiology, Dijon University Hospital, 21000 Dijon, France; ludwig.aho@chu-dijon.fr; 3 Department of Anesthesiology and critical care, St-Eloi University Hospital, 34295 Montpellier, France; s-jaber@chu-montpellier.fr; 4 Department of Hepatology, St-Eloi University Hospital, 34295 Montpellier, France; gp-pageaux@chu-montpellier.fr; 5 Department of Oncology, St-Eloi University Hospital, 34295 Montpellier, France; e-assenat@chu-montpellier.fr

**Keywords:** hepatocellular carcinoma, percutaneous thermal ablation, recurrence, survival, liver, radiofrequency ablation, microwave ablation

## Abstract

Background: To identify the predictive factors of recurrence and survival in an unselected population of Western patients who underwent multimodal percutaneous thermal ablation (PTA) for small Hepatocellular Carcinomas (HCCs). Methods: January 2015–June 2019: data on multimodal PTA for <3 cm HCC were extracted from a prospective database. Local tumor progression (LTP), intrahepatic distant recurrence (IDR), time-to-LTP, time-to-IDR, recurrence-free (RFS) and overall (OS) survival were evaluated. Results: 238 patients underwent 317 PTA sessions to treat 412 HCCs. During follow-up (median: 27.1 months), 47.1% patients had IDR and 18.5% died. LTP occurred after 13.3% of PTA. Tumor size (OR = 1.108, *p* < 0.001; hazard ratio (HR) = 1.075, *p* = 0.002) and ultrasound guidance (OR = 0.294, *p* = 0.017; HR = 0.429, *p* = 0.009) independently predicted LTP and time-to-LTP, respectively. Alpha fetoprotein (AFP) > 100 ng/mL (OR = 3.027, *p* = 0.037) and tumor size (OR = 1.06, *p* = 0.001) independently predicted IDR. Multinodular HCC (HR = 2.67, *p* < 0.001), treatment-naïve patient (HR = 0.507, *p* = 0.002) and AFP > 100 ng/mL (HR = 2.767, *p* = 0.014) independently predicted time-to-IDR. RFS was independently predicted by multinodular HCC (HR = 2.144, *p* = 0.001), treatment naivety (HR = 0.546, *p* = 0.004) and AFP > 100 ng/mL (HR = 2.437, *p* = 0.013). The American Society of Anesthesiologists (ASA) score > 2 (HR = 4.273, *p* = 0.011), AFP (HR = 1.002, *p* < 0.001), multinodular HCC (HR = 3.939, *p* = 0.003) and steatotic HCC (HR = 1.81 × 10^-16^, *p* < 0.001) independently predicted OS. Conclusions: IDR was associated with tumor aggressiveness, suggesting a metastatic mechanism. Besides AFP association with LTP, IDR, RFS and OS, treatment-naïve patients had longer RFS, and multi-nodularity was associated with shorter RFS and OS. Steatotic HCC, identified on pre-treatment MRI, independently predicted longer OS, and needs to be further explored.

## 1. Introduction

HCC is the fifth most common cancer, and the third leading cause of death from cancer worldwide [1]. Percutaneous thermal ablation (PTA) is a validated treatment option for very early and early stage HCC, together with surgical resection and liver transplantation [2]. Ultrasonography provides real-time guidance, and for that reason is the main or even the only guidance modality [3,4,5,6,7,8] for PTA of HCC. However, about 50% of planned PTAs are considered unfeasible due to invisible/inconspicuous tumor, or unsafe needle path during the planning ultrasonography [3]. Consequently, 30% of patients referred for PTA are eventually treated with palliative therapies [3], a phenomenon also noticed in real-life studies, where about 35% of patients with early HCC receive suboptimal palliative rather than curative treatments [9,10,11]. Moreover, PTA is frequently not performed in the case of high-risk HCC location (liver dome, subcapsular, near a large vessel or an adjacent organ) [12]. All of these technical issues could lead to considerable selection bias in the routine practice, thus explaining the choice of palliative treatments in many cases. Since several years, multimodality interventional suites have become available to better visualize the tumor and to allow PTA of HCC in challenging locations through the use of ultrasonography, computed tomography (CT) and angiography, alone or in combination (i.e., multimodal PTA). However, few data are available on the outcomes of multimodal PTA in unselected populations of patients with small HCC.

The risk of local tumor progression (LTP) (10%–30%) is one major concern of PTA [10]. This wide range may be explained by the considerable variety of PTA indications, techniques and imaging guidance modalities. Whether tumor size > 3 cm has been identified as a strong LTP predictor after monopolar PTA [2], some debate has emerged regarding 2–3 cm tumors [13]. Moreover, the long-term results of PTA are influenced by the high rate (up to 60%–80% at 5 years) of intrahepatic distant recurrence (IDR) [7,10,14], as observed also after HCC surgical resection [2,15]. It is unknown whether early IDR (2–3 years after PTA of < 3 cm HCC) is due to metastatic spread or de novo carcinogenesis. This is a crucial issue, particularly for developing neoadjuvant and/or adjuvant treatments, but proves difficult to assess, because basically, tumor specimens are rarely available after PTA of the primary HCC and also of the IDRs, which are frequently treated by PTA as well [16].

Therefore, the purpose of this study was to summarize the results of multimodal PTA and to identify some predictive factors of recurrence and survival in Western patients treated by multimodal PTA for small HCC.

## 2. Materials and Methods

### 2.1. Patients

This retrospective analysis was performed using data prospectively collected in a database of patients who underwent percutaneous thermal ablation (PTA) for Hepatocellular Carcinoma (HCC) at our institution. This study was approved by the Local Ethics and Research Committee (authorization number: 2017_CLER-MTP_12-04, NCT03428321 (www.clinicaltrials.gov)), and written, informed consent for the procedure and the prospective anonymized data collection was obtained from all patients during the consultation for PTA.

Inclusion criteria were: HCC diagnosed by histopathology analysis or by imaging, according to the European Association for the Study of the Liver (EASL) guidelines (23); HCC nodule(s) without extrahepatic metastases or macrovascular invasion; tumor size ≤ 30 mm; World Health Organization (WHO) performance status 0 or 1; prothrombin time (PT) ratio > 50% and platelet count higher than 5 × 10^9^/L.

Exclusion criteria were: follow-up < 2 months; Child-Pugh class B cirrhosis with a score > 7; HCC nodule adjacent to the hepatic hilum; history of biliary-digestive anastomosis or endoscopic sphincterotomy; combined treatment with embolization or chemoembolization.

Treatment was validated at a multidisciplinary meeting that included interventional radiologists, liver surgeons, oncologists, hepatologists and radiation oncologists. PTA was considered as the first-line treatment for patients with HCC stage 0 (very early stage) and stage A (early stage) according to the Barcelona Clinic Liver Cancer (BCLC) staging system [2], meaning that no technical contraindication was considered, except HCC nodule adjacent hepatic hilum.

All patients underwent contrast-enhanced multiphase computed tomography (CT) and/or magnetic resonance imaging (MRI) (including dynamic MRI) within 1 month before PTA. Laboratory tests were systematically performed to detect any possible PTA contraindication. Then, all patients were seen in consultation by the interventional radiologist.

#### Patient and Tumor Data

The following patient data were collected: age, sex, American Society of Anesthesiologists (ASA) physical status score (assessed by the anesthesiologist) (24), diabetes mellitus, liver disease (cirrhosis was defined as typical hepatic dysmorphia on imaging or by histological analysis of liver biopsy; patients were considered non-cirrhotic if their liver was defined as healthy at histology; the others were considered undetermined), steatosis (defined as signal intensity loss on opposed-phase gradient-echo sequences at baseline or at the first follow-up MRI), and Child-Pugh, MELD and ALBI scores.

The following HCC characteristics were also collected: intra-tumor steatosis (defined as a signal intensity loss on opposed-phase gradient-echo sequences) (Figure S1), dome location (i.e., when the lung parenchyma is interposed between the skin and the tumor through the anterior or lateral route on the axial plane on the baseline CT or MRI [17]), subcapsular location (i.e., direct contact with the liver capsule), adjacent to large vessels (i.e., located ≤ 5 mm from any ≥ 3 mm vessel) (29), adjacent to at-risk organs (i.e., located ≤ 5 mm from the right kidney, adrenal gland, stomach, gut or colon).

### 2.2. Percutaneous Thermal Ablation

Patients were placed in a supine position with arms above the head when possible. All procedures were done under general anesthesia with endotracheal intubation. Patients were mechanically ventilated using low tidal volume ventilation (i.e., tidal volume between 3 to 4 mL/kg, 320 mL/min minimum) to strongly limit liver movements. Respiratory rate was adjusted to maintain the end tidal carbon dioxide between 35 and 45 mmHg.

#### Guidance and Ablation

All PTAs were performed in a multimodality interventional suite. The suite was equipped with a CT scan (Optima 660, General Electric, Milwaukee, Brookfield, WI, USA) with a combined mobile C-arm (OE9900, General Electric, Milwaukee) from January 2015 to February 2017, and then with an Angio-CT Infinix-I 4D CT system (Canon Medical Systems, Tokyo, Japan) from February 2017. An ultrasound machine (Logiq E9, General Electric, Milwaukee) was available during the entire study period.

PTA was performed by four interventional radiologists (5–15 years of expertise in liver PTA) using a radiofrequency or a microwave device, depending on the operator’s choice. Radiofrequency ablations (RFAs) were performed using a clustered internally-cooled (Covidien E series, Covidien, Boulder, CO, USA) or a separable clustered internally-cooled (OctopusR, STARmed, Goyang, Korea) electrode. For this second system, one, two or three electrodes could be used. When two or three electrodes were needed, the generator (VIVA MultiR, STARmed) was in dual-switch mode. Microwave ablations (MWAs) were performed using a single 15G internally-cooled electrode and the Acculis MTA system (Angiodynamics, Amsterdam, The Netherlands). Whatever the technique, the objective was to completely ablate the tumor(s) with a 5 mm–1 cm margin. Needle track ablation was performed according to the manufacturer’s instructions.

Ultrasonography (US) guidance was the first-line guidance modality, i.e., when the tumor was visible using US, PTA was performed using US guidance. When the tumor was not visible using US or unenhanced CT, the tumor was tagged. Specifically, an angiographic catheter (4F or 5F) was inserted in the celiac trunk or the superior mesenteric artery through the femoral route. Then, a 2.7F micro-catheter (Progreat, Terumo, Tokyo, Japan) was used to reach the right or left hepatic artery (depending on the anatomical variants and HCC location).

After injection of 1.5–2 mL of lipiodol (Lipiodol ultrafluide, Guerbet, Aulnay-sous-bois, France), tumor tagging was confirmed by immediate post-injection CT. Then, CT-fluoroscopy was used to guide the PTA needle insertion in the tumor(s) (Figure S2). For tumors that are located in the liver dome and are invisible by ultrasonography, the extrapulmonary transthoracic transdiaphragmatic route was used after artificially-induced pneumothorax with CO_2_, as previously described [17,18] (Figure S3).

Hydro (5% dextrose) or CO_2_ dissection was used whenever necessary to protect the surrounding organs against thermal injury [19]. Intermittent balloon occlusion of the hepatic vein [20] was used when necessary to prevent the heat sink effect (Figure S4).

The following technical factors were systematically recorded: PTA modality (RFA or MWA), imaging guidance (ultrasonography or CT), artificial pneumothorax and tumor tagging.

Contrast-enhanced CT (portal phase) was performed immediately after the procedure to evaluate the ablation zone (i.e., the area of low attenuation) and to detect post-procedural complications. In the case of incomplete ablation or insufficient margins, the ablation needle(s) was (were) re-inserted during the same procedure to achieve complete ablation.

### 2.3. Complications

The type and number of complications were recorded and classified according to the Society of Interventional Radiology (SIR) guidelines [21]. Patients were monitored overnight. All patients underwent a clinical examination and laboratory tests the day after PTA.

### 2.4. Follow-Up and Outcome

“Technical success” was defined as complete ablation of the target tumor(s) [21] on the immediate post-PTA CT images.

Patients were followed by the same interventional radiologist who performed the PTA in consultation, immediately after the first follow-up MRI (including dynamic acquisitions) performed 4–6 weeks after the procedure, and then every 3 months.

“Treatment failure” was defined as the presence of persistent enhanced foci at the tumor site on the first follow-up MRI [22].

Complete ablation observed on first follow-up MRI was considered as the “primary treatment success”. “Secondary treatment success” was treatment success observed only after a second PTA performed within 8 weeks after the first one.

“Local tumor progression” (LTP) was any growing or enhanced tumor focus within or at the edge (direct contact) of the ablation zone, after complete ablation documented by at least one MRI [22].

“Intrahepatic distant recurrence” (IDR) was defined as any new HCC nodule in the liver, defined according to the EASL criteria [22].

### 2.5. Statistical Analysis

Normally distributed continuous variables were described with mean ± SD, non-normally distributed continuous variables with median/interquartile range (IQR), and categorical variables with numbers/percentages. Categorical variables were compared with the Fischer’s exact test, and continuous variables with the two-sided t-test or Kruskal–Wallis test, as appropriate.

The association between LTP or IDR and all patient, liver, tumor and PTA characteristics was assessed using univariate and multivariate logistic regression analyses to compute the odds ratio (OR) (with 95% confidence intervals (95% CIs)). In all cases, repeated measures logistic regression analyses were performed using a generalized estimating equation (GEE) logistic regression model to take into account PTA of several nodules and repeated PTA sessions. A robust variance estimator was used systematically. Log-linearity was checked using fractional polynomials. For the GEE model, an exchangeable correlation matrix was chosen and checked using quasi-likelihood information criteria [23].

Time-to-LTP and time-to-IDR were defined as the time from PTA to LTP and to IDR, respectively. Patients without LTP/IDR at the last follow-up/death/liver transplantation were censored at the date of the last follow-up/death/liver transplantation.

RFS was defined as the interval between PTA and death, recurrence, or last follow-up. OS was defined as the interval between PTA and death or last follow-up. Patients who underwent liver transplantation were censored at the transplantation date.

The median follow-up (95% CI) was calculated using the reverse Kaplan–Meier method. Survival curves were estimated using the Kaplan–Meier method and compared using the log-rank test. Univariate Cox proportional-hazards models of all potential baseline predictors were built to compute the hazard ratios (HRs) with their 95% CIs. Multivariate Cox models were built to include all variables found to be significant in the univariate analyses. Bootstrapping was systematically performed for internal validation (400 replications for per-tumor analyses, 200 replications for per-patient analyses).

All analyses were performed with the Stata software, version 14.0 (Stata corporation, College Station, TX, USA). A *p*-value < 0.05 was considered significant.

## 3. Results

### 3.1. Patient Characteristics

**Figure 1:**
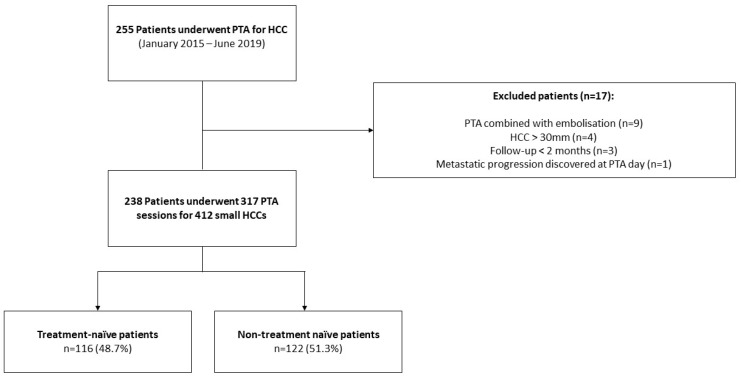
Study Flowchart.

Between January 2015 and June 2019, 255 consecutive patients underwent PTA for HCC at our institution. Seventeen patients were excluded due to combined treatment with embolization (*n* = 9), metastatic progression discovered at PTA day (*n* = 1), HCC > 30 mm (*n* = 4) and follow-up < 2 month (*n* = 3). Finally, 238 patients (median age: 64.9 ± 9.9 years; 79.8% (190/238) men) who underwent 317 PTA sessions to treat 412 HCCs were included in this study (Figure 1, and Table 1 for patient, liver disease, HCC characteristics and PTA technical factors).

### 3.2. Technical/Treatment Success

Technical success was 100%. Primary treatment success was obtained for 98.8% of nodules (407/412 HCCs). The five patients with primary treatment failure underwent a new and successful PTA procedure (100% secondary treatment success).

### 3.3. Complications and Follow-Up

In total, 15 complications (3.6%; 9/245) were observed (Table S2). No PTA-related death, needle track seeding, or liver abscess was reported. Patients were discharged the day after PTA in 94.6% (307/317) of cases.

During a median follow-up of 27.1 months (95% CI: 23.2–29.8), 18.5% (44/238) patients died and 9.2% (22/238) underwent liver transplantation.

### 3.4. Local Tumor Progression

LTP occurred after 55/412 (13.3%) ablations. The 1-year, 2-year and 3-year cumulative LTP incidence (per-tumor analysis) were 8.7%, 16.6% and 21%, respectively. The median time-to-LTP was 9.6 months (95% CI: 1.8–30). LTP occurred before IDR in 50.9% (28/55), after IDR in 20% (11/55), and concomitantly with IDR in 29.1% (16/55) of cases.

The 55 LTP were treated with PTA (*n* = 42; 76.4%), super-selective, transarterial chemoembolization (TACE) (*n* = 8, 14.5%), percutaneous ethanol injection (*n* = 1, 1.8%), stereotactic body radiation therapy (SBRT) (*n* = 2, 3.6%), or sorafenib (*n* = 1, 1.8%).

**Figure 2:**
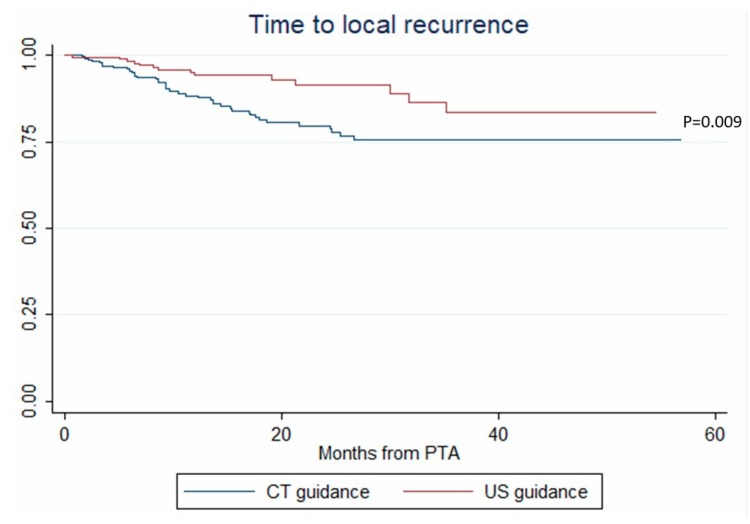
Time to local recurrence after percutaneous thermal ablation (PTA) under computed tomography (CT) and ultrasonography (US) guidance (Kaplan–Meier analysis).

In univariate analysis, age, tumor size, ultrasonography (vs. CT) guidance and tumor tagging were significantly associated with LTP (Table 2). In multivariate analysis, only tumor size (OR = 1.108, *p* < 0.001) and ultrasonography (vs. CT) guidance (OR = 0.294, *p* = 0.017) remained associated with LTP (Table 2). These results were internally validated using bootstrapping. Similarly, tumor size (HR = 1.075, *p* = 0.002) and ultrasonography (vs. CT) guidance (HR = 0.429, *p* = 0.009) independently predicted the time-to-LTP (multivariate analysis, confirmed by internal validation using bootstrapping, Data not shown) (Figure 2).

### 3.5. Intrahepatic Distant Recurrence

IDR occurred in 112/238 (47.1%) patients. The 1-year, 2-year and 3-year cumulative IRD incidences (per-patient analysis) were 33.3%, 46.9% and 63%, respectively. The median time-to-IDR was 8.4 months (95% CI: 1.6–34.3). IDR was treated with PTA (*n* = 55; 49.1%), super-selective TACE (*n* = 23, 20.5%), sorafenib (*n* = 12, 10.7%), percutaneous ethanol injection (*n* = 6; 5.4%), resection (*n* = 5; 4.5%), SBRT (*n* = 5; 4.5%), selective internal radiation therapy (*n* = 4; 3.6%) and best supportive care (*n* = 2; 1.8%).

The factors associated with IDR in univariate analysis are listed in Table 3. In multivariate analysis, alpha fetoprotein (AFP) > 100 ng/mL (OR = 3.027; *p* = 0.037) and tumor size (OR = 1.06; *p* = 0.001) were independently associated with IDR (internal validation by bootstrapping).

**Figure 3:**
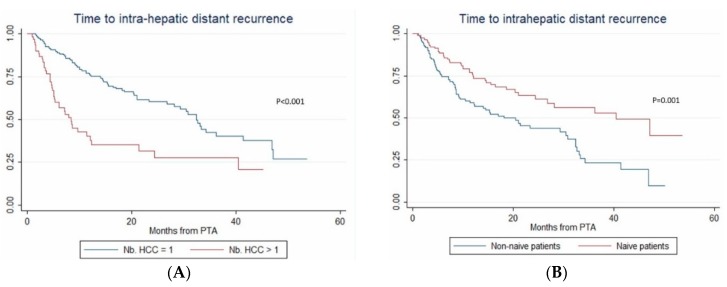

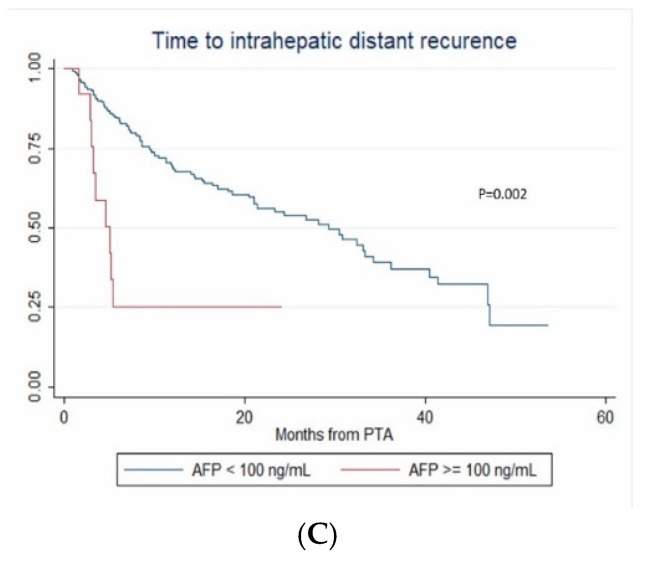
In patients with uninodular vs. multinodular HCC (**A**), in treatment-naïve vs. non-treatment naïve patients (**B**), and in patients with alpha fetoprotein (AFP) < 100 ng/mL vs. AFP ≥100ng/mL (**C**) (Kaplan–Meier analyses).

In multivariate (per-patient) analysis, multinodular HCC (HR = 2.67, *p* < 0.001), treatment-naïve patient (HR = 0.507, *p* = 0.002) and AFP > 100 ng/mL (OR = 2.767; *p* = 0.014) independently predicted time-to-IDR (confirmed after internal validation by bootstrapping, data not shown) (Figure 3). The median time-to-IDR was 40.4 vs. 18.2 months in treatment-naïve vs. non-treatment naïve patients (*p* = 0.001), 29.3 vs. 5.1 months in patients with AFP < 100 ng/mL vs. AFP ≥ 100 ng/mL (*p* = 0.002) and 32.4 vs. 8.4 months in patients with multinodular HCC vs. uninodular HCC (*p* < 0.001).

### 3.6. Recurrence-Free Survival

**Figure 4:**
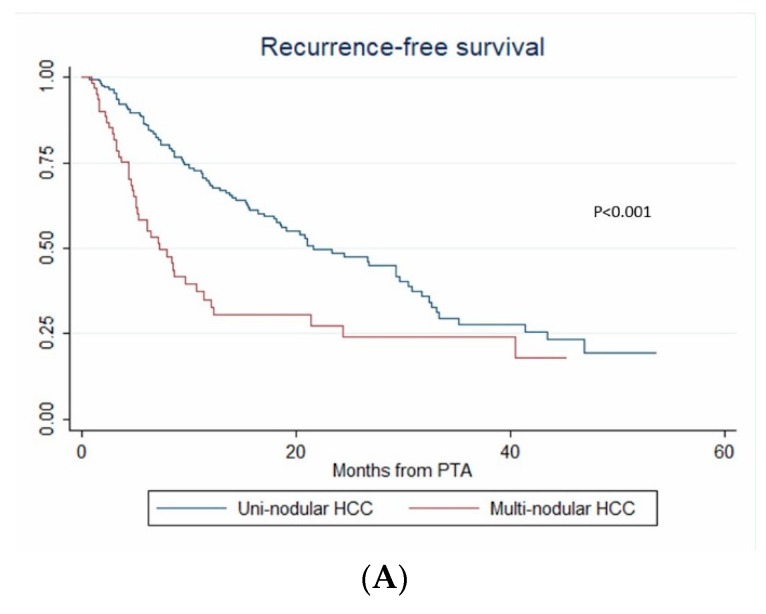

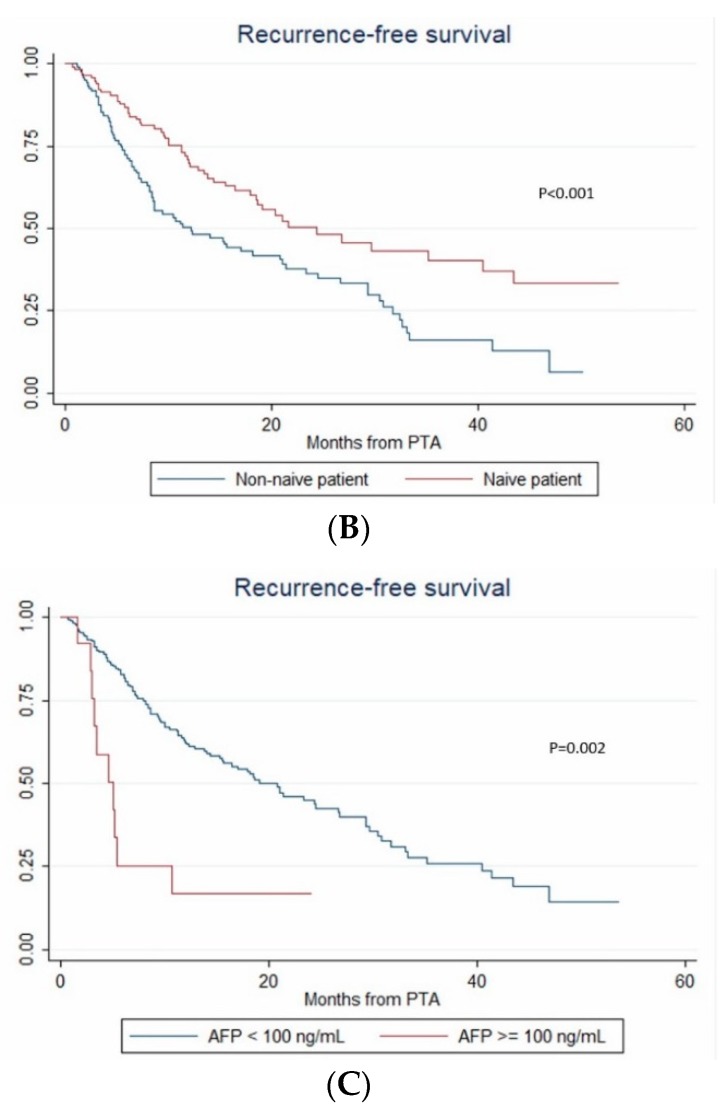
In patients with uninodular vs. multinodular HCC (**A**), in treatment naïve vs. non-treatment naïve patients (**B**), and in patients with AFP < 100 ng/mL vs. AFP ≥ 100 ng/mL (**C**) (Kaplan–Meier analyses).

During the follow-up, HCC relapsed in 52.1% (124/238) of patients. The 1-year, 2-year and 3-year RFS rates were 60.4% (95% CI: 53.5–66.6%), 46.2% (95% CI: 38.6–53.4%) and 30.3% (95% CI: 21.9–39.1%), respectively. The median RFS time was 21 months (95% CI: 15.3–26.8). Over the study period, tumor recurrence was observed in 42.3% vs. 55.4% patients with steatotic vs. non-steatotic HCC (*p* = 0.067). LTP was observed after 7.6% vs. 14.8% (*p* = 0.08) PTA of steatotic vs. non-steatotic HCC, respectively. IDR was noted in 40.4% of patients with steatotic and 48.8% of patients with non-steatotic HCC (*p* = 0.184). In univariate analysis (Table S1), multinodular HCC, treatment-naïve patient, AFP > 100 ng/mL and steatotic HCC were significantly associated with RFS. In multivariate analysis, multinodular HCC (HR = 2.144, *p* = 0.001), treatment-naïve patient (HR = 0.546, *p* = 0.004) and AFP > 100 ng/mL (HR = 2.437, *p* = 0.013) independently predicted RFS (internal validation by bootstrapping). RFS was 7.2 vs. 26.7 months in patients with multinodular HCC vs. uninodular HCC (*p* < 0.001), 26.8 vs. 12.3 months in treatment-naïve vs. non-treatment naïve patients (*p* < 0.001) and 21.4 vs. 5.1 months in patients with AFP < 100 ng/mL vs. AFP ≥ 100 ng/mL (*p* = 0.002) (Figure 4).

### 3.7. Overall Survival

Among the 44 deaths, 36.4% (16/44) were unrelated to HCC with no evidence of tumor recurrence. The 1-year, 2-year and 3-year OS rate were 98% (95% CI: 94.7–99.2%), 84.3% (95% CI: 77.5–89.2%) and 72.2% (95% CI: 62.5–79.8%), respectively (median not reached).

**Figure 5:**
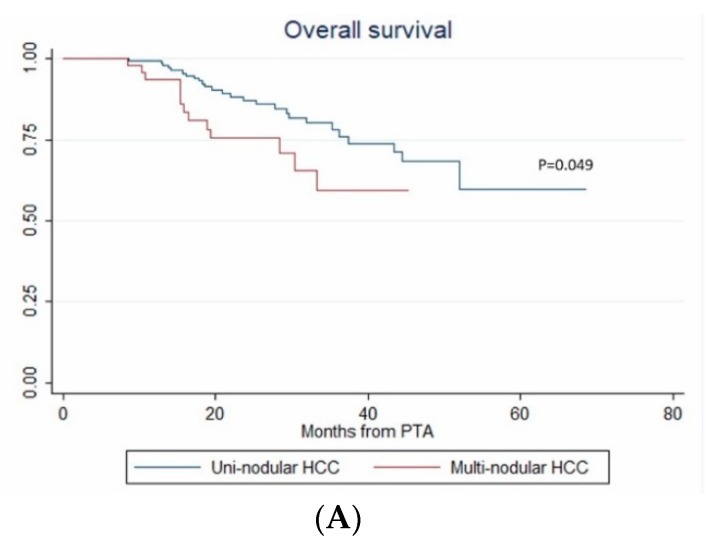

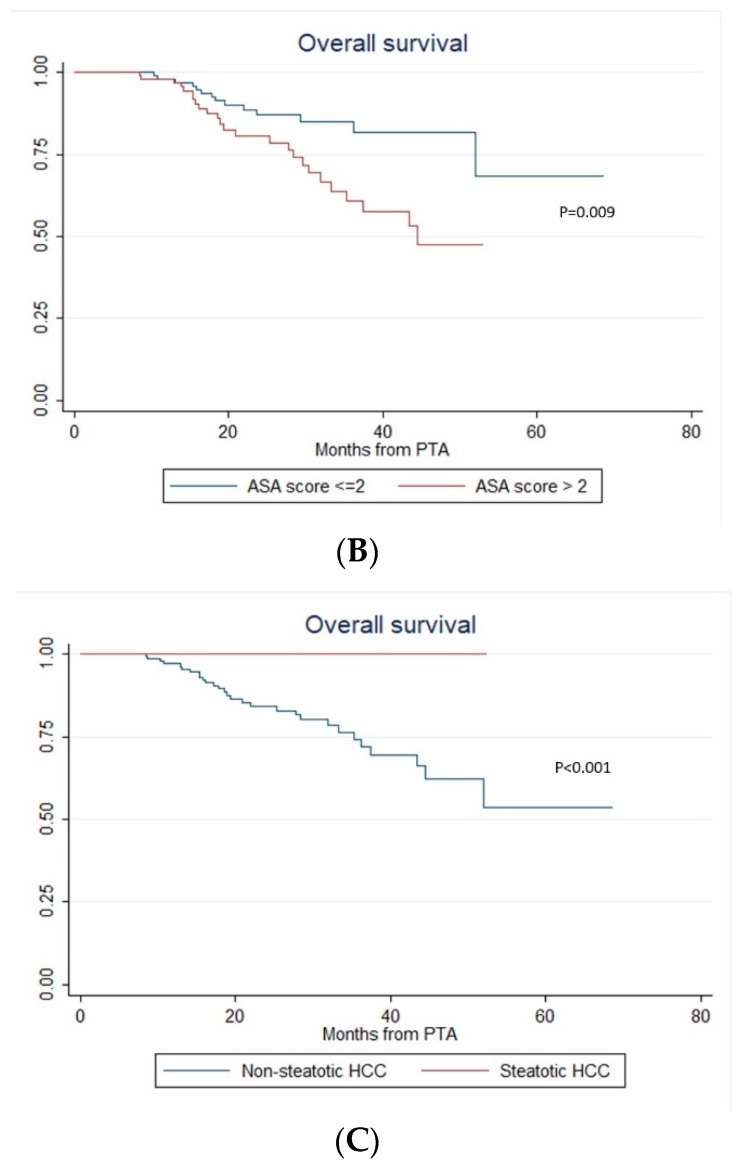
In patients with uninodular vs. multinodular HCC (**A**), with ASA score ≤ 2 vs. >2 (**B**), and with steatotic vs. non-steatotic HCC (**C**) (Kaplan–Meier analyses).

In univariate analysis, ASA score >2, MELD score > 9, AFP, multinodular HCC, steatotic HCC, PT, bilirubin, creatinine and albumin were significantly associated with OS (Table 4). In multivariate analysis, ASA score > 2 (HR = 4.273, *p* = 0.011), AFP (HR = 1.002; *p* < 0.001), multinodular HCC (HR = 3.939, *p* = 0.003) and steatotic HCC (HR = 1.81 × 10^−16^; *p* < 0.001) independently predicted OS (Figure 5). Only ASA score > 2, multinodular HCC and steatotic HCC were internally validated as independent predictors of OS (bootstrapping).

## 4. Discussion

In this study, we used a multimodal approach to perform PTA of small HCC and investigated 32 variables concerning the patient, liver disease, HCC and PTA technical factors as potential predictors of recurrence and survival. A previous study showed that ultrasonography-guided percutaneous ablation was considered not feasible in 44.5% of 898 patients during the planning ultrasonography exam, mainly because of inconspicuous HCC (72.8%) or unsafe needle path (9.5%) [24]. In a large Korean study (10,334 patients) [3], 56.8% of RFA for HCC were considered unfeasible based on the planning ultrasonography examination, and 30.3% of patients (*n* = 3132) received only palliative curative treatments. Therefore, using ultrasonography as the only imaging guidance modality for PTA induces a considerable selection bias.

Here we only considered the central HCC location as a technical contraindication and developed a multimodal strategy to address the issues of tumor poor/non-visibility or difficult location. In this study to evaluate this strategy (317 PTAs in 238 patients for 412 HCC), we reported no treatment-related death, and only 3.6% of complications, mostly managed conservatively with very limited consequences for the patients. These results are in line with previous series [10,14,25], with the notable exception that we did record any technical mistake (skin burn, gastrointestinal or colon perforation).

Moreover, treatment success (99%) and LTP (13.3%) rates compare favorably with recent studies, where LTP ranged between 13.1% and 20.5% after PTA of small HCC [3,5,8,13,26,27], especially when considering that we did not exclude patients with non/poor tumor visibility. Interestingly, CT (vs. ultrasonography) guidance was independently associated with LTP, probably because CT is not a real-time imaging modality, thus rendering more challenging the precise puncture of small HCC. We used CT guidance only in the case of poorly-/non-visible tumors by ultrasonography. Conversely, high-risk [11] or difficult tumor locations (dome, subcapsular, near adjacent organs, close to large vessels) and PTA technical factors were not independently associated with LTP, IDR, RFS or OS. Moreover, LTP was frequently (74.6%) accessible to repeated PTA sessions [10,28,29], and was not associated with OS, as shown elsewhere [3]. Therefore, multimodal PTA is a valid approach to address challenging PTAs of HCC and avoid any technical contraindication.

Tumor size is usually considered the main LTP predictive factor [2,10]. HCC < 2 cm are considered the best candidates for PTA. Conversely, some debate exists about 2–3 cm HCCs [2]. A recent study reported a 2-fold increased risk of recurrence beyond the Milan criteria in transplantable patients with > 2 cm HCC [13]. In our study (all HCC < 3 cm), tumor size > 2 cm was associated not only with a 64% increased risk of LTP, but also a 38% increased risk of IDR. In addition, even for HCC < 3cm, tumor size as a continuous variable was strongly and independently associated with LTP and IDR.

IDR occurred in 47.1% of patients (i.e., ≈ 3.5 times more frequent than LTP), in line with other studies [13,30] with comparable follow-up times, and after a median time of only 8.4 months. Distant relapse is due to tumor metastases (more frequent and with poorer prognosis) or de novo carcinogenesis [10,31,32]. Here, we investigated separately IDR occurrence and time-to-IDR. We found that variables reflecting tumor aggressiveness were the only independent predictors of IDR occurrence: AFP >100 ng/mL (3-fold increased risk of IDR) and tumor size (IDR risk increased by 6% per mm of tumor diameter increase). The temporal distribution of IDR after HCC resection [15] suggests that early relapse (within 2–3 years after PTA) is mainly related to tumor features, probably reflecting metastatic spread. In agreement, variables related to tumor aggressiveness (AFP > 100, multinodular HCC) were independently and strongly associated with shorter time-to-IDR (median time-to-IDR ≈ 6 months) in our series. These findings suggest that IDR was mainly due to metastatic spread rather than de novo carcinogenesis. In the literature, HCC recurrence has been mainly investigated in treatment-naïve patients. By using GEE models, we could incorporate in our analysis all PTAs and take into account that one patient could be treated several times. We found that the risk of IDR occurrence did not vary in treatment-naïve vs. non-treatment naïve patients. Conversely, time-to-IDR in treatment-naïve patients (i.e., first IDR occurrence) was longer (40.4 vs. 18.2 months in non-treatment-naïve patients). Not surprisingly, RFS also was significantly longer in treatment-naïve than non-treatment naïve patients (26.8 vs. 12.3 months, *p* < 0.001). Although most IDR cases were treated by curative options (mainly repeated PTA) in our series, IDR was close to statistical significance to predict OS. The high IDR rate and accumulating evidence for a metastatic mechanism encourage exploring adjuvant/neoadjuvant strategies targeting tumor growth and metastatic escape in the context of PTA for small HCC.

Not surprisingly, the serum AFP level was strongly and independently associated with both RFS and OS, as shown by other studies [4,7,33,34,35]. Conversely, limited data are available on the predictive value of the number of HCCs treated by PTA. To our knowledge, only one study compared time-to-IDR and OS in function of the number of nodules treated by PTA [4] and reported HR of 1.36 and 1.28 for time-to-IDR and OS, respectively, for multinodular (2–3) HCC compared to single tumor. This is in contrast with the strong multi-nodularity effect in our study (HR = 3.93 and 2.67 for time-to-IDR and OS, respectively). A different context (around 85% of viral infections, only 14.5% of high alcohol consumption) and more advanced HCC (29.7% of patients with >3 cm or >3 HCC nodules) [4] might explain the weaker effect of multi-nodularity in this previous study.

Surprisingly, none of the usual prognostic factors of liver disease was independently associated with RFS or OS. MELD score > 9, high bilirubin level and low albumin and PT values were associated with shorter OS in the univariate analysis. However, only a MELD score > 9 was included in the multivariate analysis (i) because of colinearity with other covariates, (ii) to prevent overfitting given the low number of events (i.e., 44 deaths) and (iii) because it had the strongest association with OS among these variables in the univariate analysis. The relatively short follow-up and the strong weight of tumor aggressiveness variables (AFP, and nodule number), or ASA score in survival models could also explain why the MELD score did not independently predict survival.

One of most striking results in this study is the independent predictive value of steatotic HCC for OS. Literature data on steatotic HCCs are very limited. In 2000, Kutami et al. [36] found that fat content in HCC at pathologic examination (51/260 HCC) was more frequent in small HCC (1.1–1.5 cm) and in the well-differentiated subtype. More recently, Chan et al. [37] showed that steatotic HCC were associated with higher metabolic risk (diabetes, arterial hypertension, steatohepatitis), smaller tumor size, lower frequency of major vessel and microvascular invasion, earlier tumor stages and lower serum AFP. In 2010, Salamao et al. first described steatohepatitic HCC, characterized by inflammatory changes, cell ballooning, peri-cellular fibrosis and Mallory–Denk bodies [38]. This variant is frequently observed in a nonalcoholic steatohepatitis (NASH) context. Analysis of the molecular and genetic alterations of steatohepatitic HCCs showed that they belong to the G4 sub-class (known to have a gene expression profile similar to that of nontumoral liver) with more favorable prognosis, they very rarely harbor the activated Wnt/beta-catenin pathway, and they lack *CTNNB1*, *TP53* and *TERT* promoter mutations [39]. In 2016, Chan et al. demonstrated that steatohepatitic HCC is a subset of steatotic HCC [37], and showed that steatotic HCC was associated with late tumor relapse after surgery [37]. In our series, RFS was longer in patients with steatotic HCC (univariate analysis), whereas LTP, time-to-LTP, IDR and time-to-IDR were not different in patients with steatotic vs. non-steatotic HCC. Again, ethnicity (Asian vs. European patients) and treatment (surgical vs. ablation) strongly differ between the study by Chan et al. and our work. Nevertheless, steatotic HCC strongly and independently predicted longer OS and none of our patients with steatotic HCC died during the follow-up. In our study, 17.2% of HCC were steatotic, like in previous series [36,37] where this definition was based on pathology findings. Chemical-shift imaging takes advantage of the difference in resonance frequency between water and fat, and is part of routine MRI protocols. It detects intratumoral steatosis with 100% specificity [40,41], allowing the noninvasive diagnosis of steatotic HCC. Accumulating evidence of a more favorable outcome strongly encourages researchers to further analyze the genomic profile of steatotic HCCs [42].

Several limitations to our study must be acknowledged. First, this is a retrospective, monocentric study, although data came from a prospectively maintained database with a strict imaging follow-up policy. We chose to include treatment-naïve and non-treatment naïve patients to explore this variable with GEE models, because a patient can be treated several times. The selection bias inherent to retrospective studies was very limited, because we included only patients with HCC nodules < 3 cm that were not centrally-located, owing to the multimodal interventional suite. Second, the median follow-up was relatively short (27.1 months). This explains the relatively low number of death events, and the limited evaluation of late HCC recurrences. Third, we could not retrospectively collect reliable information regarding abstinence, which could have influenced the overall survival of patients with alcohol-induced cirrhosis.

Then, our results need to be validated in an external series, although we systematically used bootstrapping as internal validation and to prevent overfitting [43]. Finally, like in most HCC series, the very low proportion of patients with liver biopsy did not allow exploring the tumor molecular/genetic features, especially in the case of steatotic HCC.

## 5. Conclusions

This study shows that multimodal PTA is safe and effective for treating small HCC, whatever the technical difficulty to see or to reach the tumor, and even in presumed challenging locations. CT guidance carries an increased risk of LTP, but was not associated with any survival endpoint, justifying its use as second-line guidance modality. IDR remains the most frequent event during the follow-up of patients treated by PTA for small HCC, and was associated with both tumor size and AFP, suggesting a metastatic mechanism. Besides AFP association with LTP, IDR, RFS and OS, treatment-naïve patients had longer RFS than non-treatment naïve patients, and multi-nodularity was associated with shorter RFS and OS. Finally, steatotic HCC independently predicted longer OS. This specific HCC variant, which can be identified in the pre-treatment MRI, needs to be further explored.

## Figures and Tables

**Table 1:** Characteristics of the 238 patients with 412 small Hepatocellular Carcinomas (HCCs) treated by percutaneous thermal ablation (PTA).

Characteristic	Value
**Patients**	
Age (mean ± SD, years)	64.9 ± 9.9
Sex (*n*, %)	
Male	190 (79.8)
Female	48 (20.29)
ASA score (*n*, %)	
1-2	120 (50.4)
3-4	118 (49.6)
Diabetes (*n*, %)	
No	147 (61.8)
Yes	91 (38.2)
Metformin treatment	35 (14.7)
Prior treatment for HCC (*n*, %)	
No	116 (48.7)
Yes	122 (51.3)
Resection	33 (27)
PTA	55 (45)
TACE	34 (28)
**Liver disease**	
Cirrhosis (*n*, %)	
No	20 (8.4)
Yes	218 (91.6
Causes for liver disease (*n*, %)	
Alcohol (with or without viral hepatitis)	135 (56.7)
Viral hepatitis B or C	61 (25.6)
Hemochromatosis	12 (5)
Other causes, including NASH	30 (12.6)
Steatosis (*n*, %)	
No	153 (65.7)
Yes	80 (34.3)
Child-Pugh class	
A	231 (97.1)
B	7 (2.9)
MELD score (mean ± SD)	8.8 ± 2.2
Laboratory data (mean ± SD)	
AFP (ng/mL)	36.1 ± 163.7
Total bilirubin (µmol/L)	13.5 ± 8.7
Albumin (g/L)	40.2 ± 4.9
Prothrombin activity (%)	84.6 ± 13.2
Platelet count (× 10/mm^3^)	139 ± 72
Creatinine (µmol/L)	83.7 ± 31.1
ALBI score	
1	151 (66.8)
2	75 (33.2)
**HCC**	
Method for diagnosis (*n*, %)	
Biopsy	62 (15)
Imaging	350 (85)
Tumor size (mean ± SD)	15.1 ± 5
<20 mm	358 (86.9)
>20 mm	54 (13.1)
No. of tumors (*n*, %)	
1	242 (72.9)
2	56 (21.4)
3	18 (5.4)
4	1 (0.3)
Steatotic HCC (*n*, %)	
Yes	66 (16)
No	317 (77)
Unknown	29 (7)
Dome (*n*, %)	93 (22.6)
Subcapsular location (*n*, %)	150 (36.4)
Near large vessel (*n*, %)	71 (17.2)
Near surrounding organ (*n*, %)	36 (8.7)
**PTA**	
PTA modality (*n*, %)	
Radiofrequency	174 (42.2)
Microwave	238 (57.8)
Imaging guidance (*n*, %)	
US	182 (44.2)
CT	230 (55.8)
Artificial CO_2_ pneumothorax (*n*, %)	47 (11.4)
Hydrodissection-CO_2_ dissection (*n*, %)	36 (8.7)
Tumor tagging (*n*, %)	208 (50.5)

Unless otherwise indicated, results are numbers (percentages). Abbreviations: SD, standard deviation; HCC, hepatocellular carcinoma; NASH, non-alcoholic steatohepatitis; MELD, model for end-stage liver disease; AFP, alpha fetoprotein; PTA, percutaneous thermal ablation; US, ultrasonography.

**Table 2:** Logistic regression analysis (generalized estimating equation (GEE) model) to predict local tumor progression (per tumor analysis).

Variables	Univariate Analysis		Multivariate Analysis		Bootstrapping (400 Replications)	
	Odds Ratio (95% CI)	*p* Value	Odds Ratio (95% CI)	*p* Value	Odds Ratio (95% CI)	*p* Value
**Patients**						
Age	0.968 (0.939–0.999)	**0.04**	0.969 (0.938–1.001)	0.054	0.969 (0.938–1.001)	0.058
Sex female vs male	0.487 (0.189–1.252)	0.136				
ASA (>2 vs. ≤2)	1.021 (0.565–1.845)	0.944				
Diabetes	1.665 (0.904–3.066)	0.102				
Metformin treatment	1.58 (0.6–4.19)	0.36				
Treatment-naïve patient	1.309 (0.724–2.367)	0.372				
**Liver diseases**						
Cirrhosis	4.785 (0.574–39.91)	0.148				
Child-Pugh (B vs. A)	1.559 (0.285–8.528)	0.609				
Cause of liver disease (vs. alcohol)						
Viral hepatitis B or C	0.624 (0.279–1.392)	0.249				
Hemochromatosis	0.92 (0.273–3.097)	0.893				
Others (including NASH)	0.859 (0.325–2.268)	0.758				
Steatosis	1.12 (0.607–2.064)	0.717				
AFP ≥100 vs <100 ng/mL	1.113 (0.298–4.162)	0.873				
AFP (per unit)	0.999 (0.995–1.003)	0.55				
Bilirubin	1.008 (0.977–1.04)	0.616				
Albumin	0.969 (0.909–1.032)	0.33				
Prothrombin time	1.001 (0.979–1.024)	0.922				
Platelet count (per 1,000/mm3)	1 (0.996–1.005)	0.836				
Creatinine	0.997 (0.985–1.008)	0.552				
MELD (>9 vs. ≤9)	1.263 (0.69–2.312)	0.449				
ALBI score 2 vs. 1	1.241 (0.65–2.37)	0.512				
**HCC**						
Tumor size (per mm)	1.096 (1.04–1.154)	**0.001**	1.108 (1.05–1.169)	**<0.001**	1.108 (1.051–1.168)	**<0.001**
Tumor size <20 mm	0.359 (0.181–0.709)	**0.003**				
Steatotic HCC	0.487 (0.188–1.266)	0.14				
Dome tumor	1.747 (0.942–3.24)	.077				
Subcapsular	0.932 (0.516–1.682)	0.814				
Near large vessel	1.6096 (0.809–3.202)	0.175				
Near surrounding organ	1.043 (0.387–2.809)	0.934				
**PTA**						
PTA modality: MWA vs RF	1.48 (0.81–2.703)	0.202				
US vs CT guidance	0.394 (0.208–0.749)	**0.004**	0.294 (0.107–0.803)	**0.017**	0.294 (0.102–0.841)	**0.023**
Artificial pneumothorax	1.301 (0.569–2.977)	0.533				
Tumor tagging	1.82 (1.01–3.28)	**0.046**	0.827 (0.325–2.103)	0.689	0.827 (0.362–1.887)	0.651

Abbreviations: HCC, hepatocellular carcinoma; NASH, non-alcoholic steatohepatitis; MELD, model for end-stage liver disease; AFP, alpha fetoprotein; PTA, percutaneous thermal ablation; US, ultrasonography; CT, computed tomography. Significant *p* values are marked in bold.

**Table 3:** Logistic regression analysis (GEE model) to predict intrahepatic distant tumor progression (per-tumor analysis).

Variables	Univariate Analysis		Multivariate Analysis		Bootstrapping (400 Replications)	
	Odds Ratio (95% CI)	*p* Value	Odds Ratio (95% CI)	*p* Value	Odds Ratio (95% CI)	*p* Value
**Patients**						
Age	0.98 (0.957–1.005)	0.112				
Sex female vs male	0.953 (0.526–1.727)	0.874				
ASA (>2 vs. ≤2)	0.711 (0.475–1.065)	0.098				
Diabetes	0.668 (0.394–1.131)	0.133				
Metformin treatment	0.557 (0.275–1.127)	0.104				
Treatment-naïve patient	0.999 (0.681–1.465)	0.994				
**Liver diseases**						
Cirrhosis	1.129 (0.464–2.748)	0.798				
Child-Pugh (B vs. A)	2.514 (0.668–9.459)	0.173				
Cause of liver disease (vs. alcohol)						
Viral hepatitis B or C	1.067 (0.605–1.88)	0.823				
Hemochromatosis	0.955 (0.321–2.847)	0.934				
Others (including NASH)	0.753 (0.351–1.618)	0.468				
Steatosis	1.205 (0.76–1.91)	0.428				
AFP ≥ 100 vs < 100 ng/mL	3.576 (1.224–10.441)	**0.02**	3.027 (1.068–8.576)	**0.037**	3.027 (1.032–8.876)	**0.044**
AFP (per unit)	1.001 (0.999–1.002)	0.396				
Bilirubin	0.993 (0.968–1.018)	0.581				
Albumin	0.955 (0.914–0.998)	**0.04**	0.966 (0.923–1.011)	0.139	0.966 (0.896–1.042)	0.37
Prothrombin time	0.996 (0.98–1.012)	0.668				
Platelet count (per 1000/mm^3^)	1 (0.997–1.003)	0.952				
Creatinine	0.992 (0.984–1.002)	0.087				
MELD (>9 vs. ≤9)	0.756 (0.493–1.161)	0.202				
ALBI score 2 vs. 1	1.406 (0.909–2.175)	0.125				
**HCC**						
Tumor size (per mm)	1.053 (1.02–1.087)	**0.002**	1.06 (1.025–1.097)	**0.001**	1.06 (1.014–1.108)	**0.01**
Tumor size < 20 mm	0.615 (0.388–0.975)	0.039				
Steatotic HCC	0.714 (0.431–1.184)	0.192				
Dome tumor	0.736 (0.502–1.082)	0.12				
Subcapsular	0.991 (0.713–1.376)	0.955				
Near large vessel	0.973 (0.613–1.544)	0.908				
Near surrounding organ	1.22 (0.696–2.138)	0.488				
**PTA**						
PTA modality: MWA vs RF	1.363 (0.956–1.943)	0.087				
US vs CT guidance	1.006 (0.712–1.423)	0.973				
Artificial pneumothorax	1.301 (0.569–2.977)	0.533				
Tumor tagging	0.864 (.616–1.212)	0.399				

Abbreviations: HCC, hepatocellular carcinoma; NASH, non-alcoholic steatohepatitis; MELD, model for end-stage liver disease; AFP, alpha fetoprotein; PTA, percutaneous thermal ablation; US, ultrasonography; CT, computed tomography. Significant *p* values are marked in bold.

**Table 4:** Univariate and multivariate Cox regression models to predict overall survival (per patient analysis).

Variables	Univariate Analysis		Multivariate Analysis		Bootstrapping (200 Replications)	
	Odds Ratio (95% CI)	*p* Value	Odds Ratio (95% CI)	*p* Value	Odds Ratio (95%CI)	*p* Value
**Patients**						
Age	1.012 (0.978–1.046)	0.496				
Sex female vs. male	0.431 (0.150–1.238)	0.118				
ASA (>2 vs. ≤2)	2.404 (1.248–4.628)	**0.009**	4.273 (1.386–13.171)	0.011	4.273 (1.097–16.646)	0.036
Diabetes	1.563 (0.839–2.912)	0.16				
Metformin treatment	1.092 (0.434–2.752)	0.851				
Treatment-naïve patient	0.692 (0.361–1.326)	0.267				
Local recurrence	1.016 (0.515–2.004)	0.964				
IDR	1.97 (0.988–3.931)	0.054				
**Liver diseases**						
Cirrhosis	1.397 (0.312–6.258)	0.662				
Child-Pugh (B vs. A)	1.003 (0.094–10.707)	0.998				
Cause of liver disease (vs. alcohol)						
Viral hepatitis B or C	0.994 (0.493–2.005)	0.986				
Hemochromatosis	0.407 (0.062–2.671)	0.349				
Others (including NASH)	0.537 (0.177–1.631)	0.273				
Steatosis	1.255 (0.654–2.406)	0.495				
AFP ≥ 100 vs < 100 ng/mL	4.435 (1.456–13.513)	**0.009**				
AFP (per unit)	1.0012 (1.001–1.003)	**<0.001**	1.002 (1.001–1.003)	**<0.001**	1.002 (0.998–1.006)	0.293
Bilirubin	1.039 (1.002–1.077)	**0.038**				
Albumin	0.915 (0.86–0.978)	**0.009**				
Prothrombin time	0.973 (0.949–0.998)	**0.031**				
Platelet count (per 1,000/mm3)	0.999 (0.994–1.003)	0.615				
Creatinine	1.007 (1.002–1.011)	**0.003**				
MELD (>9 vs. ≤9)	2.361 (1.253–4.448)	**0.008**	2.014 (0.772–5.255)	0.153	2.014 (0.669–6.063)	0.213
ALBI score 2 vs. 1	1.675 (0.869–3.23)	0.123				
**HCC**						
Tumor size (per mm)	1.023 (0.975–1.074)	0.35				
Tumor size < 20 mm	1.159 (0.498–2.697)	0.731				
Nb. of HCC (1 vs. >1)	1.979 (1.003–3.903)	**0.049**	3.939 (1.601–9.69)	**0.003**	3.939 (1.198–12.947)	**0.024**
Steatotic HCC	4.15x10^-16^ (2.44 × 10^-16^–7.07 × 10^-16^)	**<0.001**	1.81 × 10^-16^ (7.96 × 10^-17^–4.13 × 10^-16^)	**<0.001**	1.81x10^-16^ (5.47 × 10^-20^–6.02 × 10^-13^)	**<0.001**
Dome tumor	1.152 (0.575–2.307)	0.691				
Subcapsular	0.813 (0.41–1.609)	0.552				
Near large vessel	1.334 (0.683–2.605)	0.399				
Near surrounding organ	0.404 (0.09–1.822)	0.238				
**PTA**						
PTA modality: MWA vs RF	1.19 (0.845–1.69)	0.08				
US vs CT guidance	0.797 (0.418–1.517)	0.489				
Artificial pneumothorax	1.301 (0.569–2.977)	0.533				
Tumor tagging	0.774 (0.4–1.499)	0.448				

Abbreviations: IDR, intra-hepatic distant recurrence; HCC, hepatocellular carcinoma; NASH, non-alcoholic steatohepatitis; MELD, model for end-stage liver disease; AFP, alpha fetoprotein; PTA, percutaneous thermal ablation; US, ultrasonography; CT, computed tomography. Significant *p* values are marked in bold.

## Supplementary Materials

The following are available online at https://www.mdpi.com/2072-6694/12/2/313/s1, Figure S1: In-phase (A) and opposed-phase (B) chemical-shift imaging showing signal intensity loss on the opposed-phase image (arrow) in a typical steatotic HCC, Figure S2 Intra-arterial injection of lipiodol to tag a HCC invisible by ultrasonography (A). Then, the HCC becomes hyperdense and a microwave ablation needle is inserted under CT-guidance (B and C), Figure S3 After intra-arterial injection of lipiodol to tag a HCC located under the liver dome (thus invisible by ultrasonography), pneumothorax was artificially induced with CO_2_ using a Veress needle (A) and the radiofrequency-ablation needle was inserted through the extrapulmonary transthoracic transdiaphragmatic route (B), Figure S4 Three radiofrequency ablation needles were inserted in a HCC nodule located close to the middle hepatic vein (A). Then, an 11mm-balloon was inflated to stop the blood flow in the middle hepatic vein in order to prevent the heat sink effect (B). Table S1: Table S1. Univariate and multivariate Cox regression models to predict recurrence-free survival (per patient analysis), Table S2. Complications observed after 412 PTA sessions.
