# Supplementary material for: Multimodal Percutaneous Thermal Ablation of Small Hepatocellular Carcinoma: Predictive Factors of Recurrence and Survival in Western Patients
Source: Cancers (Basel). 2020 Jan 29;12(2):313. doi: 10.3390/cancers12020313 (PMC7072144; doi:10.3390/cancers12020313)
Supplement: Supplementary file 1 [file cancers-12-00313-s001.pdf]

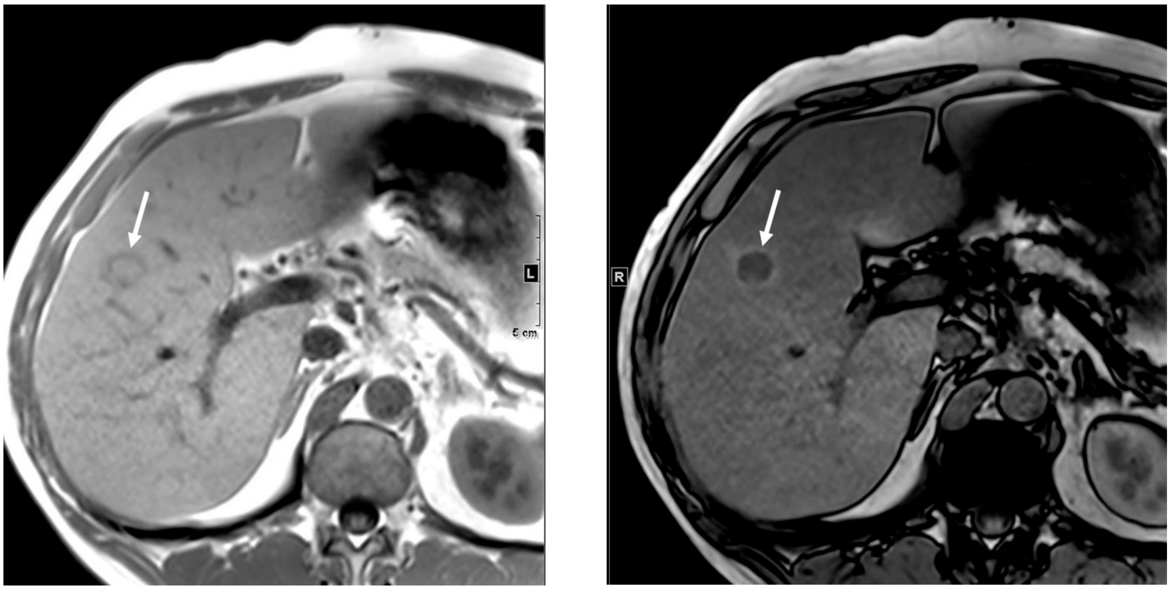

A B

Figure S1 In-phase (A) and opposed-phase (B) chemical-shift imaging showing signal intensity loss on the opposed-phase image (arrow) in a typical steatotic HCC.

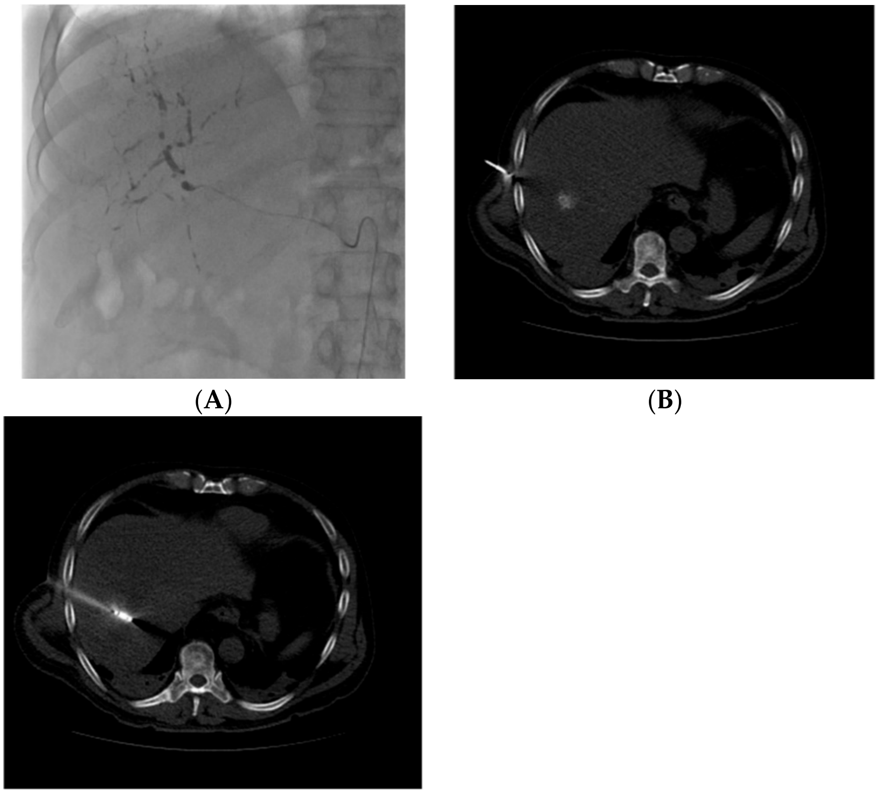

(**A**) (**B**) (**C**)

Figure S2 Intra-arterial injection of lipiodol to tag a HCC invisible by ultrasonography (A). Then, the HCC becomes hyperdense and a microwave ablation needle is inserted under CT-guidance (B and C).

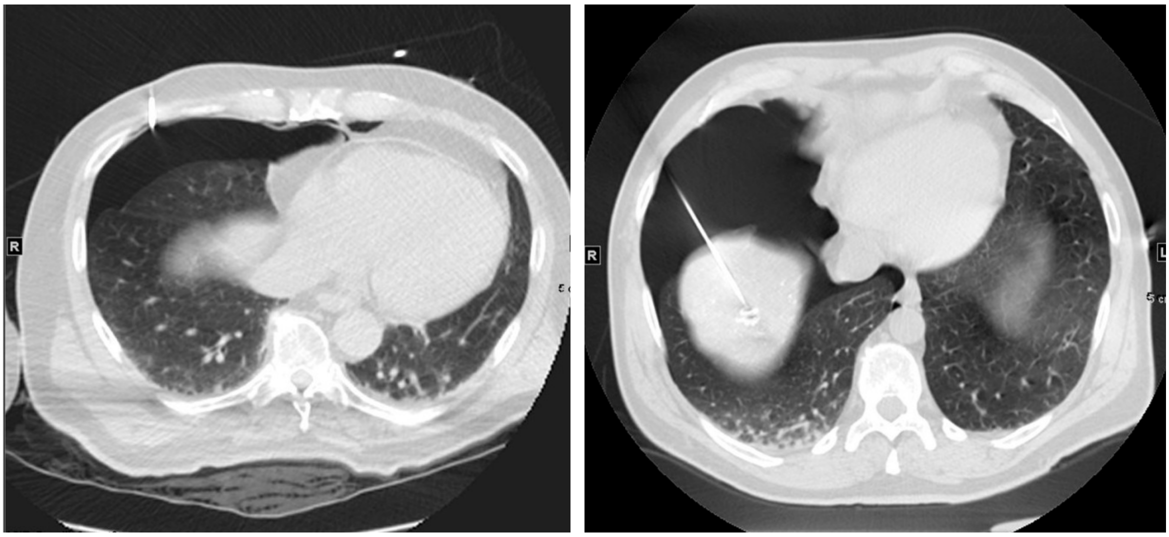

(**A**) (**B**)

Figure S3 After intra-arterial injection of lipiodol to tag a HCC located under the liver dome (thus invisible by ultrasonography), pneumothorax was artificially induced with CO_2 using a Veress needle (A) and the radiofrequency-ablation needle was inserted through the extrapulmonary transthoracic transdiaphragmatic route (B).

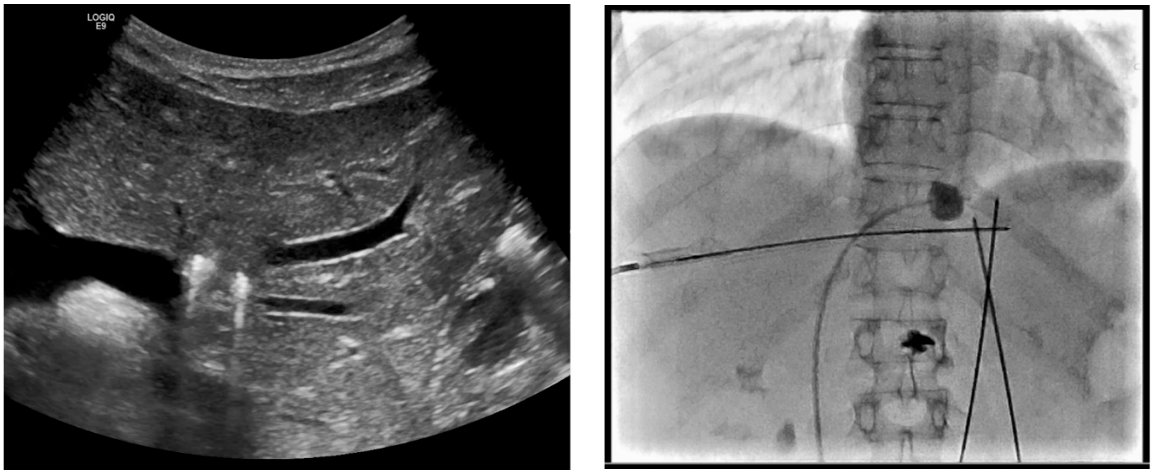

(**A**) (**B**)

Figure S4 Three radiofrequency ablation needles were inserted in a HCC nodule located close to the middle hepatic vein (A). Then, an 11mm-balloon was inflated to stop the blood flow in the middle hepatic vein in order to prevent the heat sink effect (B).

Supplemental tables

Table S1. Univariate and multivariate Cox regression models to predict recurrence-free survival (per patient analysis).

	Univariate analysis	Multivariate analysis	Bootstrapping (200 replications)

**Variables**	**Odds ratio (95% CI)**	**P value**	Odds ratio (95% CI)	P value	Odds ratio (95%CI)	P value
**Patients**						
**Age**	1.006 (0.987-1.024)	0.551				
**Sex female vs male**	0.862 (0.555-1.339)	0.51				
**ASA (>2 vs. ≤2)**	0.855 (0.608-1.2)	0.365				
**Diabetes**	0.887 (0.628 – 1.252)	0.495				
**Metformin treatment**	0.813 (0.468-1.41)	0.46				
**Treatment-naïve patient**	0.548 (0.387-10.776)	**0.001**	0.546 (0.362-0.823)	**0.004**	0.546 (0.353-0.844)	**0.006**
**Liver diseases**						
**Cirrhosis**	1.082 (0.506-2.313)	0.839				
**Child-Pugh (B vs. A)**	1.584 (0.821-3.055)	0.17				
**Cause of liver disease (vs. alcohol)**						
**Viral hepatitis B or C**	0.895 (0.590-1.358)	0.601				
**Hemochromatosis**	1.178 (.519-2.674)	0.696				
**Others (including NASH)**	0.741 (0.453-1.213)	0.233				
**Steatosis**	1.188 (0.83-1.7)	0.346				
**AFP ≥100 vs <100 ng/mL**	3.349 (1.469-7.637)	**0.004**	2.437 (1.211-4.906)	**0.013**	2.437 (1.129-5.264)	**0.023**
**AFP (per unit)**	1.001 (1 – 1.001)	**0.006**				
**Bilirubin**	1.01 (0.989-1.032)	0.344				
**Albumin**	0.984 (0.953-1.017)	0.338				
**Prothrombin time**	1 (0.987-1.013)	0.970				
**Platelet count (per 1,000/mm3)**	1 (0.998-1.002)	0.897				
**Creatinine**	1 (0.995-1.004)	0.872				
**MELD (>9 vs. ≤9)**	1.105 (0.78-1.567)	0.573				
**ALBI score 2 vs. 1**	1.16 (0.815-1.65)	0.41				
**HCC**						
**Tumor size (per mm)**	1.02 (0.99-1.051)	0.19				
**Tumor size <20 mm**	0.835 (0.569-1.225)	0.356				
**Nb. of HCC (1 vs. >1)**	2.102 (1.382-3.197)	**0.001**	2.144 (1.357-3.388)	**0.001**	2.144 (1.298-3.543)	**0.003**
**Steatotic HCC**	0.61 (0.389-0.955)	**0.031**	0.864 (0.540-1.383)	0.544	0.864 (0.495-1.509)	0.608
**Dome tumor**	1.053 (0.717-1.547)	0.791				
**Subcapsular**	0.964 (0.673-1.379)	0.839				
**Near large vessel**	1.01 (0.686-1.487)	0.959				
**Near surrounding organ**	0.88 (0.483-1.604)	0.677				
**PTA**						
**PTA modality: MWA vs RF**	1.196 (0.845-1.693)	0.087				
**US vs CT guidance**	0.938 (0.669-1.316)	0.712				
**Artificial pneumothorax**	1.301 (0.569-2.977)	0.533				
**Tumor tagging**	0.97 (0.688-1.369)	0.863				

Abbreviations: HCC, hepatocellular carcinoma; NASH, non-alcoholic steatohepatitis; MELD, model for end-stage liver disease; AFP, alpha fetoprotein; PTA, percutaneous thermal ablation; US, ultrasonography; CT, computed tomography.

Table S2. Complications observed after 412 PTA sessions.

Grade	Complication	Nb.
**Minor complications (1.7%) (grade B SIR)**	Hemoperitoneum without active bleeding (no embolization)	4
	Liver subcapsular hematoma without consequences	1
	Hemothorax without consequence	1
	Hepatic vein thrombosis	1
**Major complications (1.9%) (grade C SIR)**	Hemoperitoneum with active bleeding on post-procedure CT and embolization	2
	Pleural fistula requiring drainage	1
	Pneumothorax requiring drainage for 24h	5
